# MRI-based evaluation of orbital structures in relation to thyroid function in thyroid eye disease

**DOI:** 10.1007/s12020-026-04625-4

**Published:** 2026-05-02

**Authors:** Tugba Barlas, Mehmet Muhittin Yalcin, Esra Temel, Emetullah Cindil, Bercin Tarlan, Onur Konuk, Goksun Ayvaz, Fusun Balos Toruner

**Affiliations:** 1 Faculty of Medicine, Department of Endocrinology and Metabolism, Gazi University, Ankara, Turkey; 2 Faculty of Medicine, Department of Radiology, Gazi University, Ankara, Turkey; 3 Faculty of Medicine, Department of Ophthalmology, Gazi University, Ankara, Turkey; 4 Department of Endocrinology and Metabolism, Koru Hospital, Ankara, Turkey

**Keywords:** Euthyroid Graves’ disease, Hashimoto thyroiditis, Orbita magnetic resonance imaging, Thyroid eye disease, Thyroid stimulating immunoglobulin

## Abstract

**Aim:**

Although most patients with thyroid eye disease (TED) present with hyperthyroidism, some remain euthyroid or hypothyroid throughout long-term follow-up. We aimed to evaluate TED using MRI-based orbital measurements and investigate the influence of thyroid function on proptosis, extraocular muscles (EOM) and retrobulbar fat edema.

**Methods:**

Patients with TED who underwent orbital MRI were included. Patients were categorized as hyperthyroid or euthyroid/hypothyroid based on thyroid function. The total EOM thickness was calculated as the sum of the diameters of the EOMs. Proptosis was evaluated by measuring the distance between the anterior globe and the interzygomatic line. T2 signal intensity and retrobulbar fat edema were assessed, with edema graded on a 0–3 scale.

**Results:**

Seventy-four patients were included: 59(79.7%) with hyperthyroid and 15(20.3%) with euthyroid/hypothyroid. The groups did not differ in sex, age, or clinical activity score (CAS)(*p* > 0.05). The hyperthyroid group showed greater anterior globe–interzygomatic line distance(*p* < 0.05), while retrobulbar fat edema was more pronounced in the euthyroid/hypothyroid TED group(*p* < 0.05). Total EOM thickness, asymmetry, and T2 signal heterogeneity did not differ significantly between groups(*p* > 0.05). In the regression model, total EOM thickness was independently associated with the highest thyroid-stimulating immunoglobulin (TSI) value of patients (β = 0.335,*p* = 0.045), whereas asymmetric EOM involvement, CAS, proptosis, T2 signal intensity, and retrobulbar fat edema showed no significant associations.

**Conclusion:**

Although the hyperthyroid and euthyroid/hypothyroid TED groups differed in proptosis and retrobulbar fat edema, the total EOM thickness and asymmetric involvement did not differ significantly. Total EOM thickness was associated with the highest TSI values after covariate adjustment.

**Supplementary Information:**

The online version contains supplementary material available at 10.1007/s12020-026-04625-4.

## Introduction

Thyroid eye disease (TED) also known as thyroid-associated ophthalmopathy is characterized by enlargement of the extraocular muscles and an increase in orbital fat volume [1]. Although the exact mechanism remains unclear, it is thought that antibodies against the thyroid-stimulating hormone (TSH) receptor interact with antigens in the orbital tissues, leading to infiltration by T lymphocytes and subsequent release of inflammatory mediators [2, 3].

Although Graves’ disease is the most common underlying cause, cases associated with Hashimoto thyroiditis have also been reported [4–6]. In TED, thyroid hormone levels and thyrotropin receptor antibodies (TRAb) are typically elevated. However, a small number of patients remain euthyroid without developing hyperthyroidism during long-term follow-up [7]. Muñoz-Ortiz et al. reported the prevalence of euthyroidism, hypothyroidism, and hyperthyroidism in patients with TED as 7.9%, 10.36%, and 86.2%, respectively [8]. Euthyroid Graves’ disease (EGD) is a rare clinical entity defined as infiltrative orbitopathy occurring in the absence of current or previous biochemical thyroid abnormalities and without any history of antithyroid therapy [7, 9]. Due to its low prevalence and incidence, diagnosis is challenging. As EGD might represent an early stage of thyroid dysfunction, repeated evaluation of thyroid function is essential during follow-up [7]. Moreover, it is possible that a subgroup of euthyroid patients might have experienced undiagnosed transient episodes of thyroid dysfunction [10]. A limited number of studies in the literature examining euthyroid or hypothyroid TED have highlighted potential differences compared to hyperthyroid TED with conflicting results. Clinical activity tends to be milder, and the disease has been reported to present with a higher degree of asymmetry [7, 11–13].

Orbital magnetic resonance imaging (MRI) offers significant advantages in the objective and comprehensive evaluation of TED due to its high soft tissue contrast, lack of radiation exposure, and ability to provide detailed anatomical and structural assessment of the extraocular muscles and retrobulbar fat [14]. These features make it a valuable diagnostic tool for identifying chronic structural changes and variations associated with thyroid function. In this context, it was aimed to evaluate TED using MRI-based orbital measurements and investigate the influence of thyroid function on proptosis, extraocular muscles (EOM) and retrobulbar fat edema in the present study.

## Materials and methods

### Study design and Subjects

This study was conducted retrospectively at a tertiary referral center between 2015 and 2023. The study adhered to the principles of the Declaration of Helsinki and received approval from Gazi University Ethical Committee (Ethics approval number:396). Due to the retrospective nature of the study, the requirement for written informed consent was waived. All patient data were anonymized prior to analysis to ensure privacy and confidentiality.

Ophthalmic manifestations of TED were assessed based on standardized EUGOGO guidelines [15]. The inclusion criteria were: (i) age over 18 years, (ii) being under follow-up for TED, (iii) having undergone orbital MRI, and (iv) having available thyroid-stimulating antibody (TSI) levels. The exclusion criteria comprised the following: (i) patients without orbital MRI evaluation, (ii) unavailable thyroid-stimulating immunoglobulin (TSI) levels, (iii) presence of a non-thyroidal secondary cause that could lead to ophthalmopathy, and (iv) follow-up duration of less than 12 months. All eligible patients who met the predefined inclusion criteria were consecutively recruited during the study period.

Patients were classified into two groups based solely on their thyroid function status before or within 12 months after the onset of TED. The hypothyroid/euthyroid TED group included patients who had normal thyroid function (euthyroid) or were diagnosed with overt or subclinical hypothyroidism and did not develop hyperthyroidism during follow-up. The hyperthyroid TED group included patients who had been diagnosed with hyperthyroidism either before or within 12 months after the onset of TED, regardless of whether they subsequently achieved euthyroid or hypothyroid status after treatment. Patients who developed hypothyroidism as a consequence of hyperthyroidism treatment were not included in the hypothyroid/euthyroid TED group.

### Clinical and Laboratory Assessment

Clinical activity was evaluated using the Clinical Activity Score (CAS). A CAS score ≥ 3 was classifed as an active disease [16]. Smoking status was defined as current smoking at the time of MRI evaluation. Thyroid function was evaluated by measuring serum levels of free triiodothyronine (FT3), free thyroxine (FT4), and thyroid-stimulating hormone (TSH). Serum TSI levels were determined using a chemiluminescent immunoassay method with the Immulite system (Siemens Healthcare Diagnostics, USA).

### MRI Assessment

The measurements were performed on archived magnetic resonance images. All image measurements were performed by a single experienced radiologist and subsequently reviewed by a second experienced radiologist for accuracy and consistency. Orbital MR images of the patients included in the study were obtained using 3T MRI systems with dedicated head coils, depending on the imaging center. The primary imaging protocol at our institution was performed on a 3T MRI system (Magnetom Verio, Siemens Healthcare, Erlangen, Germany) and included sagittal and coronal T2-weighted turbo spin-echo sequences (repetition time [TR] ≈ 4200 ms, echo time [TE] ≈ 107 ms), axial and coronal T2-weighted fat-suppressed turbo spin-echo sequences (TR ≈ 3660 ms, TE ≈ 101 ms), sagittal T1-weighted turbo spin-echo images (TR ≈ 400 ms, TE ≈ 40 ms), and axial and coronal T1-weighted fat-suppressed turbo spin-echo sequences (TR ≈ 650 ms, TE ≈ 10 ms), and axial diffusion-weighted imaging with b-values of 0 and 1000 s/mm². A limited number of MRI examinations were obtained from external institutions using routine clinical orbital MRI protocols. Across centers, orbital MRI protocols consistently included standard T1- and T2-weighted sequences acquired in axial and coronal planes, with comparable spatial resolution suitable for EOM measurements. Typical imaging parameters across centers included a matrix size of approximately 256 × 256, a field of view of approximately 160 mm, a slice thickness of approximately 3 mm, and a minimal interslice gap. The following orbital measurements were obtained:

- To assess proptosis; the distance between the interzygomatic line and the anterior globe was measured on axial images.
- The thickness of the EOM was evaluated by measuring the coronal MRI sections obtained perpendicular to the long axis of the muscles. The diameter of each EOM was measured at its point of maximum thickness, which was defined as the slice demonstrating the largest cross-sectional area. The superior rectus and levator palpebrae superioris muscles were evaluated in a collective manner as the superior muscle group, and their combined diameter was measured at the level immediately before the separation of the intervening fat plane.
- The total EOM thickness was calculated as the sum of the diameters of the EOMs, including the medial rectus, lateral rectus, superior muscle group and inferior rectus [17].
- T2 signal intensity was evaluated using non–fat-suppressed T2-weighted coronal sequences, which allowed for visual assessment of intramuscular signal changes. The signal pattern of each muscle was categorized as either homogeneous or heterogeneous.
- Edema in the retrobulbar compartments was assessed using fat-suppressed T2-weighted images. Signal intensity was scored semi-quantitatively by the radiologist on a scale from 0 to 3.

In this study, EOM thicknesses were assessed based on the population-based reference values reported by Özgen et al. to evaluate muscle enlargement [18]. Additionally, proptosis was evaluated using the distance between the interzygomatic line and the anterior globe, with age- and sex-specific normative values from the literature [19].

Asymmetric involvement was defined based on established radiological criteria [20]. Muscle asymmetry was considered present when the right-to-left diameter ratio of any EOM exceeded 1.4 [21]. In addition, a proptosis difference greater than 2 mm between the two eyes was accepted as evidence of asymmetric proptosis [22, 23].

### Statistical Analysis

Statistical analyses were conducted using SPSS version 22.0 and R version 4.3.2 (https://www.r-project.org). The Shapiro-Wilk test was applied to assess whether continuous data followed a normal distribution. Normally distributed continuous variables were expressed as the mean ± standard deviation (SD), while non-normally distributed variables were presented as the median and interquartile range (25th–75th percentile, IQR). Continuous variables were compared using the Mann-Whitney U test or the independent samples t-test. Categorical variables were compared using the chi-squared test or Fisher’s exact test. Bonferroni correction was applied to adjust for multiple testing in exploratory analyses involving multiple extraocular muscle thickness parameters. Analyses of covariance (ANCOVA) were used to assess group-level differences in imaging parameters according to thyroid functional status, adjusting for age, sex, smoking status, and prior TED-related treatments when applicable. A multivariate logistic regression analysis was conducted to identify independent predictors of heterogeneity in T2 signal intensity on MRI. Furthermore, a linear regression analysis was performed to explore the factors associated with the highest TSI value. The dependent variable was log-transformed to meet the normality assumption for linear regression analyses. The statistical significance level was determined as *p* < 0.05.

## Results

A total of 74 patients were included in the study. Of these, 59 patients (79.7%) were classified within the hyperthyroid TED group, and 15 patients (20.3%) were in the hypothyroid/euthyroid TED group. The clinical and laboratory findings of the groups are summarized in Table 1. In the hyperthyroid TED group, TSI positivity was more frequent (*p* < 0.001), whereas both the positivity (*p* = 0.031) and titer of anti-TPO (*p* = 0.008) were higher in the hypothyroid/euthyroid TED group.

**Table 1:** Clinical and laboratory assessment of patients with TED

	Hyperthyroid (*n* = 59)	Hypothyroid/ Euthyroid (*n* = 15)	*P*-value
Age, years	46.3 ± 11.6	50.8 ± 12.7	0.195
Sex, female, n (%)	43 (72.9)	13 (86.7)	0.267
CAS ≥ 3, n (%)	22 (37.3)	3 (20.0)	0.206
TSI positivity, n (%)	58 (98.3)	7 (46.7)	**< 0.001**
Anti-TPO positivity, n (%)	24 (40.7)	11 (73.3)	**0.031**
Anti-TPO (IU/mL)	7.0 (0.7–186.0)	450.0 (29.7–816.5)	**0.008**
Anti-Tg (IU/mL)	0.9 (0.9–1.8)	80.5 (0.6–239.2)	0.137
Current smoker, n (%)	32 (55.4)	7 (46.7)	0.600

TED: Thyroid eye disease, CAS: Clinical activity score, TSI: Thyroid-stimulating immunoglobulin, Anti-TPO: Anti-thyroid peroxidase antibody, Anti-Tg: Anti-thyroglobulin antibody, IU/mL: International units per milliliter

Statistically significant parameters are given in bold

**Fig. 1:**
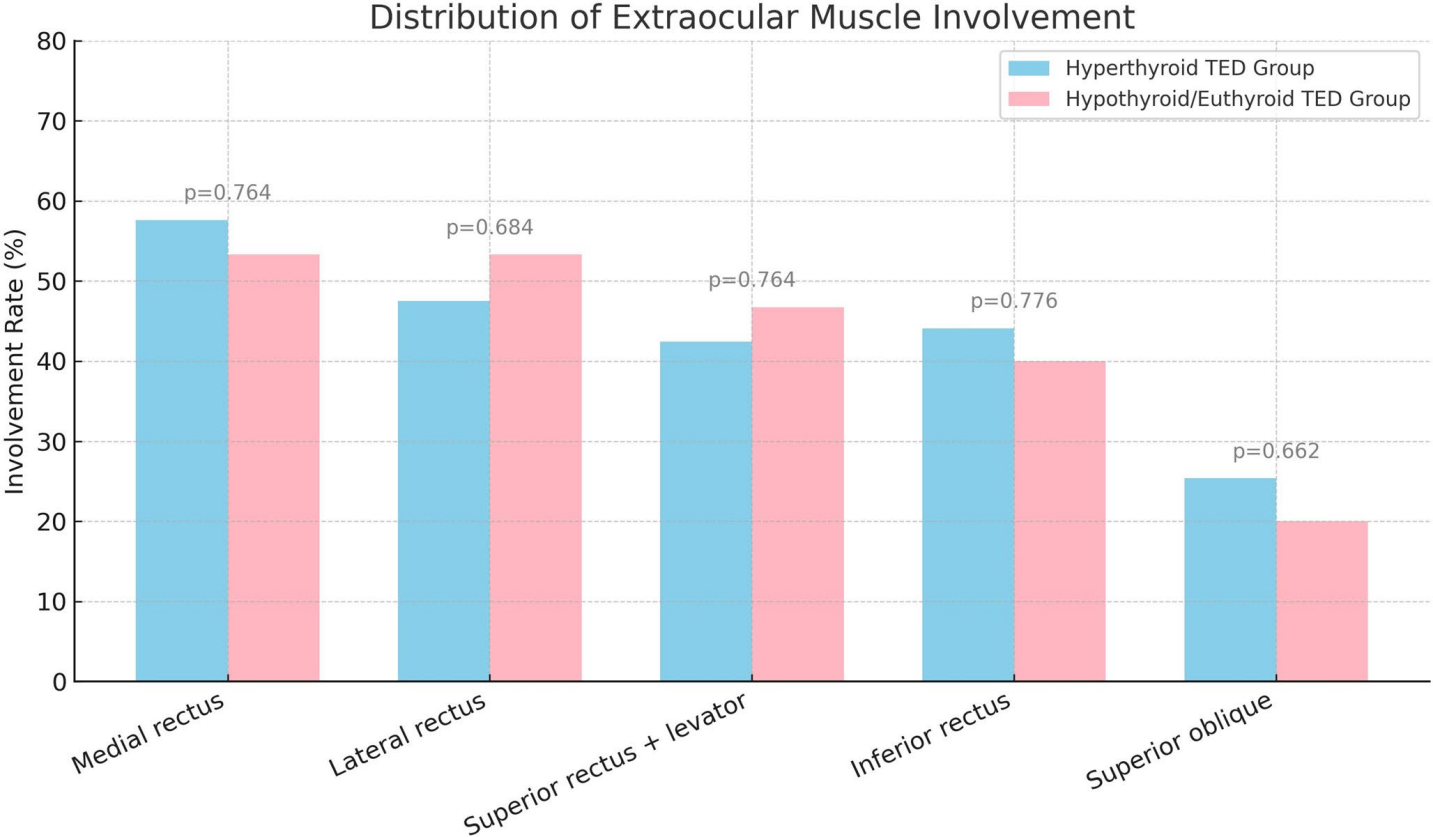
Distribution of extraocular muscle involvement in patients with TED TED: Thyroid eye disease

**Fig. 2:**
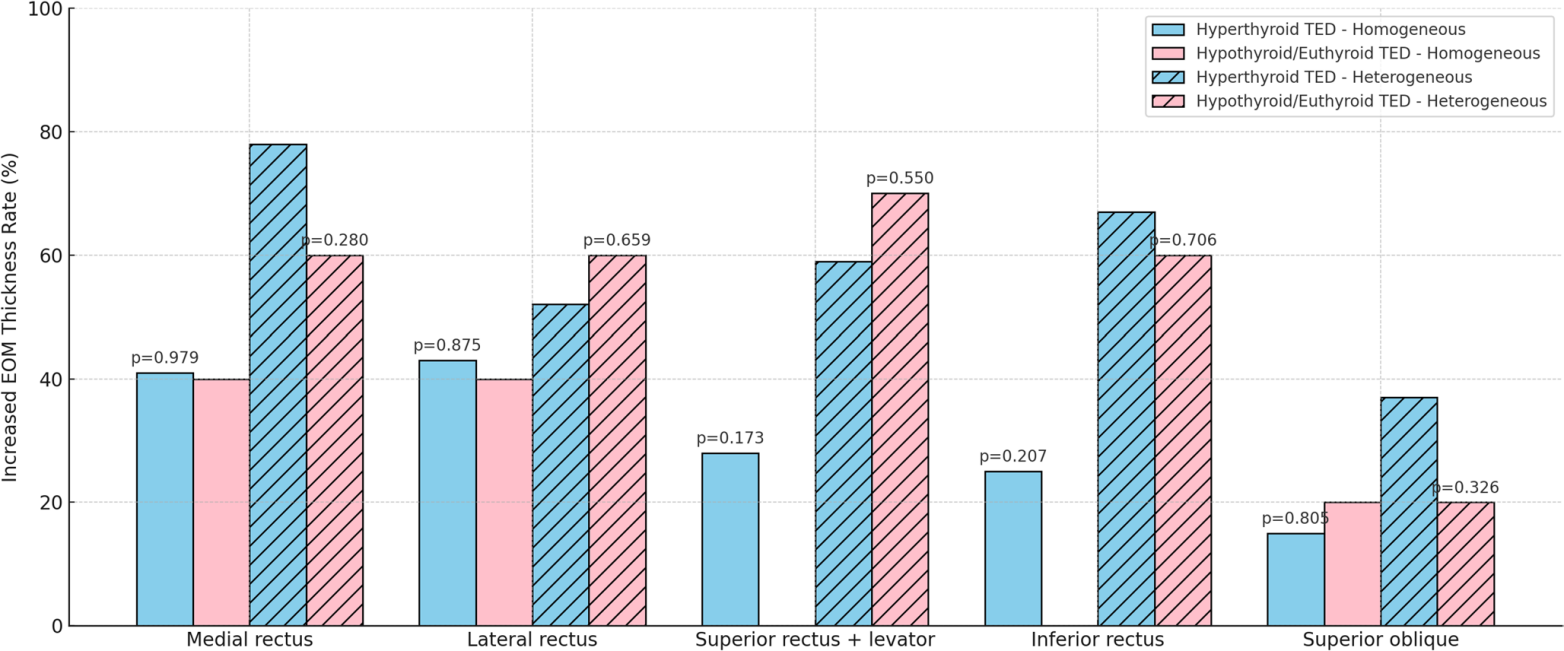
Distribution of extraocular muscle involvement in patients with TED according to T2 signal intensity TED: Thyroid eye disease

In the hypothyroid/euthyroid TED group, the anterior globe–interzygomatic line distance was measured as 18.7 (16.7–21.5) mm across 30 eyes of 15 patients. In contrast, it was 21.7 (18.6–24.7) mm in 118 eyes of 59 patients in the hyperthyroid group (*p* = 0.003). After adjustment for age, sex, and smoking status using ANCOVA, thyroid functional status remained independently associated with anterior globe–interzygomatic line distance (*p* = 0.011). Comparisons of right and left eye measurements between the hyperthyroid group and the euthyroid/hypothyroid group are presented in Table 2. No significant difference was observed between the groups in terms of asymmetric proptosis (*p* = 0.720) (Table 2). The EOM thicknesses of the hyperthyroid and hypothyroid/euthyroid TED groups are provided in the Supplementary Table. There were no significant differences between the groups in terms of individual extraocular muscle thicknesses or total EOM thickness (*p* > 0.05). Median total EOM thickness was 39.9 mm (IQR, 32.0–51.5 mm) in the hyperthyroid TED group and 38.6 mm (IQR, 35.8–64.0 mm) in the hypothyroid/euthyroid TED group (*p* = 0.256). Figure 1 illustrates the involvement rates of each EOM. No significant difference was observed between the hyperthyroid and hypothyroid/euthyroid TED groups regarding the presence of asymmetric involvement based on EOM involvement (*p* = 0.603). Heterogeneous T2 signal intensity of the EOMs was observed in 45.8% of the hyperthyroid TED group and 66.7% of the hypothyroid/euthyroid TED group (*p* = 0.148). When the increase in EOM thickness was evaluated separately in patients with homogeneous and heterogeneous T2 signal intensity, no significant difference was observed between the hyperthyroid and hypothyroid/euthyroid TED groups (Fig. 2).

**Table 2:** Assessment of patients with TED in terms of proptosis

	Hyperthyroid (*n* = 59)	Hypothyroid/ Euthyroid (*n* = 15)	*p*-value
Anterior globe–interzygomatic line / Right (mm)	21.8 ± 4.5	19.3 ± 3.3	**0.029 **
Anterior globe–interzygomatic line / Left (mm)	21.6 ± 4.4	19.1 ± 3.3	**0.026**
Proptosis, n (%)	50 (84.7)	9 (60.0)	**0.033**
Asymmetric proptosis, n (%)	14/50 (28.0)	2/9 (22.2)	0.720

TED: Thyroid eye disease

The rate of asymmetric proptosis was reported only in patients with proptosis

Statistically significant parameters are given in bold

Data on previous TED-related treatments were available for 63 patients. Prior corticosteroid therapy was administered to 6 patients (40.0%) in the hypothyroid/euthyroid TED group and to 23 patients (46.0%) in the hyperthyroid TED group (*p* = 0.591), while prior orbital radiotherapy was performed in 2 patients (13.3%) and 1 patient (1.7%), respectively (*p* = 0.138). Patients with prior corticosteroid therapy exhibited significantly greater thickness of the right inferior rectus (*p* = 0.007), left inferior rectus (*p* = 0.032), and total EOM (*p* = 0.049) compared with those without previous corticosteroid treatment in the overall group. No significant association was observed between EOM thickness and prior orbital radiotherapy (*p* > 0.05). An ANCOVA model including age, sex, smoking status, prior corticosteroid therapy, and orbital radiotherapy as covariates showed that there was no significant difference in total EOM thickness between the hyperthyroid and euthyroid/hypothyroid TED groups after adjustment (*p* = 0.460).

**Fig. 3:**
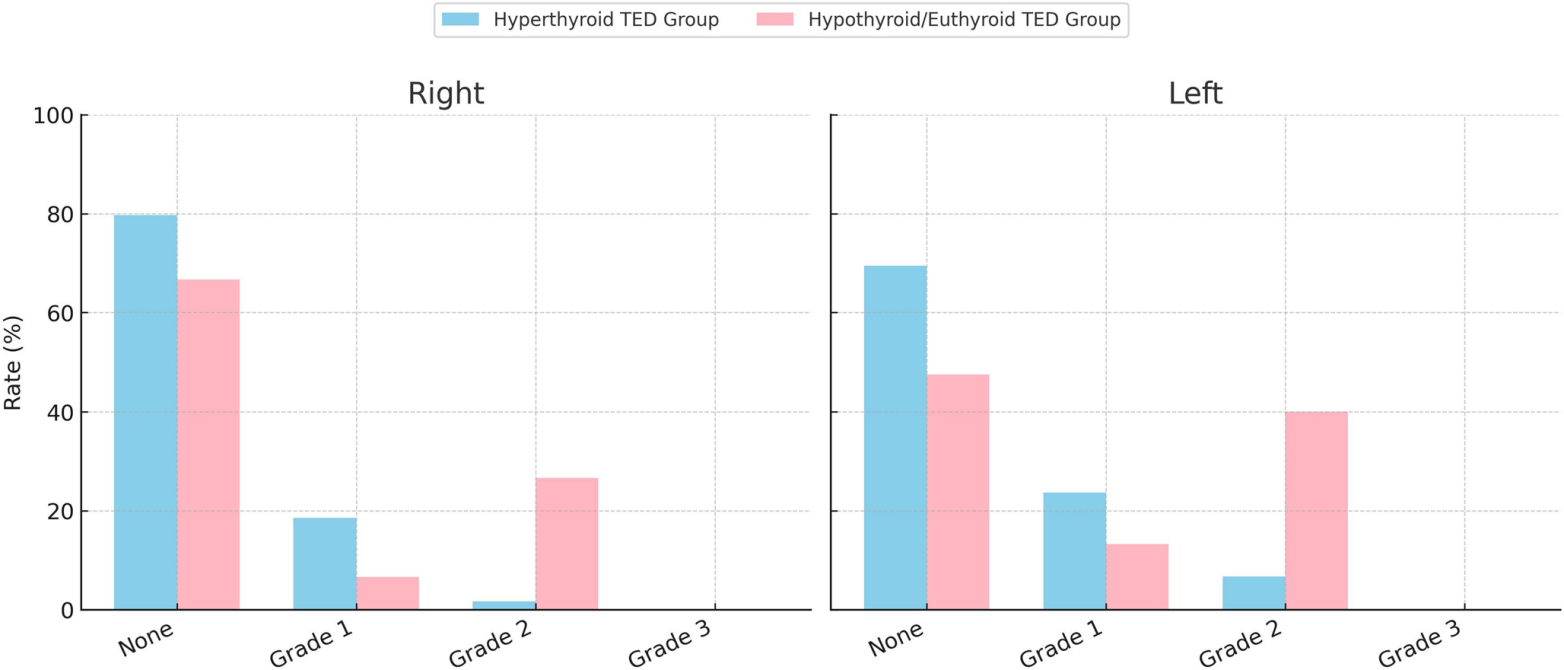
Evaluation of retrobulbar fat edema in patients with TED TED: Thyroid eye disease

In the hyperthyroid TED group, right orbital edema was observed as grade 0 in 79.7% of patients, grade 1 in 18.6%, and grade 2 in 1.7%, whereas in the hypothyroid/euthyroid TED group, the corresponding rates were 66.7%, 6.7%, and 26.7%, respectively (*p* = 0.002). Regarding left orbital edema, 69.5% of patients in the hyperthyroid TED group had grade 0, 23.7% had grade 1, and 6.8% had grade 2, while in the hypothyroid/euthyroid TED group, the rates were 47.6%, 13.3%, and 40.0%, respectively (*p* = 0.027). No patients exhibited grade 3 edema (Fig. 3). There was no significant association between the presence of retrobulbar fat edema and the CAS of patients (*p* = 0.288).

In the multiple linear regression model with the highest TSI value of patients as the dependent variable, total EOM thickness was independently associated with higher TSI values (β = 0.335, *p* = 0.045). None of the other covariates, including asymmetric involvement of the EOMs, CAS, proptosis, T2 signal intensity, or retrobulbar fat edema, showed a statistically significant association (Table 3). Furthermore, in multivariate logistic regression analysis, both smoking (OR 3.87, 95% CI 1.22–12.30, *p* = 0.021) and retrobulbar fat edema (OR 3.91, 95% CI 1.12–13.61, *p* = 0.032) were identified as independent predictors of heterogeneity in T2 signal intensity on MRI (Table 4).

**Table 3:** Linear regression analysis of factors associated with the highest TSI value

Variable	B coefficient	95% CI for B	*P* value
Total EOM thickness	0.017	0.000–0.033	**0.045**
CAS	0.068	-0.091–0.227	0.397
Proptosis	-0.108	-0.679–0.463	0.706
T2 signal intensity on MRI	0.137	-0.325–0.599	0.554
Orbital fat edema	-0.149	-0.594–0.296	0.504
Asymmetric EOM involvement	0.378	-0.058–0.814	0.088

TSI: Thyroid-stimulating immunoglobulin, EOM: Extraocular muscles, CAS: Clinical activity score

Dependent variable: log10-transformed TSI (log10TSI)

Statistically significant parameters are given in bold

The median interval between the highest recorded TSI value and orbital MRI was 2 months (IQR: 0–13 months)

**Table 4:** Logistic regression analysis of parameters associated with heterogeneity in T2 signal intensity on MRI

Variable	S.E.	Exp(B)	95% CI	*P* value
Current smoker, yes/no	0.588	3.871	1.22–12.30	**0.021**
Orbital fat edema, yes/no	0.634	3.907	1.12–13.61	**0.032**
TSI positivity, yes/no	0.917	3.204	0.53–19.26	0.204
Anti-TPO, IU/mL	0.001	1.001	0.999–1.003	0.341
CAS ≥ 3, yes/no	0.610	2.409	0.73–7.97	0.150

MRI: Magnetic resonance imaging, TSI: Thyroid-stimulating immunoglobulin, Anti-TPO: Anti-thyroid peroxidase antibody, CAS: Clinical activity score

Dependent variable: T2 signal intensity on MRI

Statistically significant parameters are given in bold

## Discussion

In our study, 20.3% of patients with TED exhibited a hypothyroid or euthyroid course. A recent review evaluating the distribution of thyroid functional status in TED reported hypothyroidism in 10.36% of patients and euthyroidism in 7.9% [8]. MRI is widely recognized as an essential tool for the differential diagnosis of patients with hypothyroid or euthyroid presentations [7]. Therefore, the inclusion of only patients who underwent orbital MRI in our cohort may explain the higher incidence observed compared with previously reported data. Indeed, another study from our center including all patients with TED reported that the thyroid function distribution at the initial endocrinology visit was 5.8% hypothyroid and 9.6% euthyroid between 1998 and 2007, and 4.9% hypothyroid and 9.3% euthyroid between 2008 and 2017 [24].

Although the existing literature comprises a limited number of studies investigating TED in patients with hypothyroid or euthyroid status, these studies primarily focus on the clinical activity of the disease [12, 13, 25, 26]. It was reported that patients with euthyroid or hypothyroid TED develop milder symptoms, exhibit lower CAS scores, and present with more asymmetrical disease compared with hyperthyroid patients [12, 13]. Differently, our study focused on chronic structural changes by evaluating orbital MRI findings. Although proptosis was more pronounced in hyperthyroid patients, no difference was observed between hyperthyroid and hypothyroid/euthyroid TED patients regarding asymmetric involvement. The lack of a significant difference in asymmetric involvement may reflect the similar levels of inflammatory activity, as indicated by comparable CAS values between the groups. In addition, unlike the previously mentioned studies that relied on Hertel exophthalmometry, we employed objective MRI assessment using the interzygomatic line–anterior globe distance and predefined asymmetry criteria, which may also account for this finding. Variations in baseline orbital anatomy across different racial and ethnic groups may further influence the assessment of proptosis and asymmetric involvement. In this context, the role of standardized imaging becomes particularly relevant. Recent multicenter investigations and clinical guidelines have increasingly emphasized the impact of ethnicity-related anatomical variations in orbital structure on both disease presentation and radiologic interpretation in TED [27, 28]. These findings further support the use of orbital MRI to enhance diagnostic precision and improve disease activity assessment across diverse populations, thereby contributing to more personalized and consistent management approaches.

We did not observe any difference in either the rate of EOM thickening or total EOM thickness between hyperthyroid and hypothyroid/euthyroid TED patients. Previous studies have reported that heterogeneity of the EOMs on T2-weighted MRI and the presence of low signal intensity are associated with limited treatment response and suggest early fibrotic changes [29]. The short-tau inversion-recovery (STIR) MRI technique is also considered useful for evaluating inflammation in the EOMs in TED, as edematous inflammatory lesions typically demonstrate high signal intensity on STIR images [30]. However, in the present study, T2 signal characteristics were assessed qualitatively as homogeneous or heterogeneous, and a quantitative signal intensity index could not be calculated due to variability in MRI scanners and acquisition parameters associated with the retrospective design. Given that MRI examinations may have been performed at different stages of the disease, a subgroup analysis was conducted focusing on patients with heterogeneous T2 signal characteristics, assuming that this group might represent a more comparable disease phase. Notably, even within this subgroup, no difference in the rate of increased EOM thickness was observed between hyperthyroid and hypothyroid/euthyroid TED patients.

Retrobulbar fat edema on MRI did not correlate with CAS in our study. In literature, prior studies have conflicting findings, while some report an association with CAS [31], Kaichi et al. [32] found no significant correlation between the orbital fat water fraction and CAS. This difference may reflect that CAS assesses anterior soft-tissue inflammatory signs, whereas MRI detects deep orbital fat involvement. The faster resolution of clinical signs compared with radiologic changes, phenotype heterogeneity (fat-predominant disease), and CAS measurement limitations might also attenuate associations.

The prevalence of TED in hyperthyroidism is well recognized because of its high frequency. In contrast, its lower prevalence in hypothyroid and euthyroid patients often leads to their exclusion from evaluation, which can delay diagnosis and treatment. The main strength of our study is the detailed assessment of chronic structural changes in this patient group using orbital MRI findings. This study also has several limitations. First, this was a single-center, retrospective study with a relatively limited number of patients. Nevertheless, as our institution serves as a referral center for TED, a noteworthy sample size was achieved in this relatively rare patient group. The imbalance between thyroid functional subgroups reflects the natural distribution of thyroid functional status in TED, particularly given the lower prevalence of euthyroid and hypothyroid TED in routine clinical practice. This imbalance may have limited the ability to detect subtle differences between groups. Second, despite a thorough diagnostic work-up for TED, the presence of alternative etiologies cannot be completely excluded in some patients within the euthyroid group in the absence of tissue diagnosis. In addition, image measurements were performed by a single experienced radiologist and subsequently reviewed by a second radiologist; however, formal inter-observer agreement metrics were not calculated due to the lack of independent duplicate measurements. The consistency of our findings with previously reported data from the Turkish population [18] supports the reliability of the measurement approach. Due to the retrospective design of the study, MRI examinations may have been performed at different stages of TED. Functional assessments were not consistently performed at the same time as MRI, precluding reliable structure–function analyses. Moreover, although a certain level of standardization was achieved by including orbital MRIs obtained with comparable spatial resolution and standard T1- and T2-weighted sequences across centers, differences in MRI scanners and acquisition parameters prevented quantitative evaluation of T2 signal intensity. Future prospective studies using standardized MRI protocols may provide a more detailed evaluation of these imaging features and improve their clinical applicability.

## Conclusion

Our findings demonstrate that MRI-based orbital measurements offer valuable insights into the characteristics of euthyroid and hypothyroid TED. While significant differences were observed between the hyperthyroid and euthyroid/hypothyroid groups in terms of proptosis and retrobulbar fat edema, the total EOM thickness and asymmetric involvement were comparable between the groups. Furthermore, total EOM thickness was associated with the highest TSI values after covariate adjustment. 

## Supplementary Information

Below is the link to the electronic supplementary material.

Supplementary Material 1

## Data Availability

The datasets generated and/or analysed during the current study are available from the corresponding author on reasonable request.
