# Supplementary material for: MRI-based evaluation of orbital structures in relation to thyroid function in thyroid eye disease
Source: Endocrine. 2026 May 2;91(1):155. doi: 10.1007/s12020-026-04625-4 (PMC13134976; doi:10.1007/s12020-026-04625-4)
Supplement: Supplementary file 1 — Supplementary Material 1 [file 12020_2026_4625_MOESM1_ESM.docx]

**Supplementary Table.** The extraocular muscle thicknesses of the hyperthyroid and hypothyroid/euthyroid TED groups

|  | Hyperthyroid TED  (n=59) | Hypothyroid/Euthyroid TED  (n=15) | P  value |
| --- | --- | --- | --- |
| Medial rectus/right | 4.8 (4.0–6.4) | 5.1 (4.3–8.2) | 0.142 |
| Medial rectus/left | 5.1 (4.1–6.3) | 4.9 (4.5–7.9) | 0.472 |
| Lateral rectus/right | 4.5 ± 1.9 | 4.9 ± 1.7 | 0.398 |
| Lateral rectus/left | 4.3 ± 1.9 | 5.4 ± 1.7 | 0.047 |
| Superior rectus + levator/right | 5.7 ± 1.9 | 5.4 ± 2.0 | 0.629 |
| Superior rectus + levator/left | 5.0 (4.3–6.1) | 5.4 (4.6–7.0) | 0.310 |
| Inferior rectus/right | 5.2 (4.2–7.0) | 5.3 (4.8–8.7) | 0.229 |
| Inferior rectus/left | 5.4 (4.4–7.3) | 5.4 (4.7–8.5) | 0.339 |
| Superior oblique/right | 3.4 (2.8–4.1) | 3.0 (3.0–3.5) | 0.492 |
| Superior oblique/left | 3.3 (2.6–3.8) | 2.9 (2.7–3.2) | 0.058 |
| Inferior oblique/right | 3.2 (2.6–3.8) | 3.2 (2.7–3.4) | 0.349 |
| Inferior oblique/left | 3.2 (2.7–3.8) | 3.4 (2.8–4.3) | 0.336 |

TED: Thyroid eye disease

Bonferroni correction was applied for multiple comparisons. Considering 12 exploratory comparisons, the adjusted threshold for statistical significance was set at p < 0.004.
